# Sensitization of U937 leukemia cells to doxorubicin by the MG132 proteasome inhibitor induces an increase in apoptosis by suppressing NF-kappa B and mitochondrial membrane potential loss

**DOI:** 10.1186/1475-2867-14-13

**Published:** 2014-02-04

**Authors:** Pablo César Ortiz-Lazareno, Alejandro Bravo-Cuellar, José Manuel Lerma-Díaz, Luis Felipe Jave-Suárez, Adriana Aguilar-Lemarroy, Jorge Ramiro Domínguez-Rodríguez, Oscar González-Ramella, Ruth De Célis, Paulina Gómez-Lomelí, Georgina Hernández-Flores

**Affiliations:** 1 División de Inmunología, Centro de Investigación Biomédica de Occidente (CIBO), Instituto Mexicano del Seguro Social (IMSS), Guadalajara, Jalisco, México; 2 Centro Universitario de los Altos, Universidad de Guadalajara (UdeG), Tepatitlán de Morelos, Jalisco, México; 3 Departamento de Farmacobiología, Centro Universitario de Ciencias Exactas e Ingeniería, UdeG, Jalisco, México; 4 Servicio de Hemato-Oncología Pediatría, OPD Hospital Civil Juan I. Menchaca, Guadalajara, Jalisco, México; 5 Programa de Doctorado en Ciencias Biomédicas Orientación Inmunología, Centro Universitario de Ciencias de la Salud, UdeG, Jalisco, México

**Keywords:** Apoptosis, NF-кB, Caspase activation, Mitochondrial dysfunction, Senescence, MG132, Doxorubicin, Leukemia

## Abstract

**Background:**

The resistance of cancerous cells to chemotherapy remains the main limitation for cancer treatment at present. Doxorubicin (DOX) is a potent antitumor drug that activates the ubiquitin-proteasome system, but unfortunately it also activates the Nuclear factor kappa B (NF-кB) pathway leading to the promotion of tumor cell survival. MG132 is a drug that inhibits I kappa B degradation by the proteasome-avoiding activation of NF-кB. In this work, we studied the sensitizing effect of the MG132 proteasome inhibitor on the antitumor activity of DOX.

**Methods:**

U937 human leukemia cells were treated with MG132, DOX, or both drugs. We evaluated proliferation, viability, apoptosis, caspase-3, -8, and −9 activity and cleavage, cytochrome *c* release, mitochondrial membrane potential, the Bcl-2 and Bcl-XL antiapoptotic proteins, senescence, *p65* phosphorylation, and pro- and antiapoptotic genes.

**Results:**

The greatest apoptosis percentage in U937 cells was obtained with a combination of MG132 + DOX. Likewise, employing both drugs, we observed a decrease in tumor cell proliferation and important caspase-3 activation, as well as mitochondrial membrane potential loss. Therefore, MG132 decreases senescence, *p65* phosphorylation, and the DOX-induced Bcl-2 antiapoptotic protein. The MG132 + DOX treatment induced upregulation of proapoptotic genes *BAX*, *DIABLO*, *NOXA*, *DR4*, and *FAS*. It also induced downregulation of the antiapoptotic genes *BCL-XL* and *SURVIVIN.*

**Conclusion:**

MG132 sensitizes U937 leukemia cells to DOX-induced apoptosis, increasing its anti-leukemic effectiveness.

## Background

Leukemias are a heterogenic group of diseases characterized by infiltration of the neoplastic cells of the hematopoietic system into the blood, bone marrow, and other tissues. They represent the most common cancer in children and the leading cause of infant death from cancer worldwide [1,2]. Generally, this type of tumor is treated with chemotherapy, but its effectiveness is sometimes limited by the generation of drug resistance as well as by multiple side effects [3-6]. In this regard, Doxorubicin (DOX) is a chemotherapeutic agent that belongs to the anthracycline possessing family; its effectiveness has been well-documented in clinical and experimental protocols against liquid and solid tumors. Several studies have demonstrated that DOX can induce apoptosis *in vivo* and *in vitro* in several types of tumor cells [7-10].

Major efforts are being conducted to identify the mechanisms underlying tumor resistance to anticancer drugs. In this regard, DOX can activate the ubiquitin-proteasome system that regulates the Nuclear factor kappa-B (NF-кB) transcription factor, which promote proliferation and survival in tumor cells [11,12]; thus, overactivation of NF-кB has been shown in several tumor types [13]. MG132 is a proteasome inhibitor that induces apoptosis in tumor cells [14]; the combination of proteasome inhibitors with some antitumor drugs comprises a new emerging field in Oncology [15,16]. It has been demonstrated that the MG132 proteasome inhibitor can interrupt the NF-кB pathway [17]. Under normal conditions, this factor is linked with its specific inhibitor I kappa B (IкB). Chemo- and radiotherapy can induce the phosphorylation of IкB; then, this molecule is degraded in the proteasome and the phosphorylated NF-кB is able to translocate to the nucleus, activating genes involved in tumor cell proliferation and survival [18,19]. In fact, sensitivity to chemotherapy is determined by genes (*BCL-2* and *BCL-XL)* that regulate the apoptotic process [20]. The expression of these antiapoptotic proteins is in turn regulated by NF-кB [21]. The balance of pro- and antiapoptotic proteins is an important determinant of cell sensitivity to apoptosis [22]. Chemotherapeutic agents such as DOX exhibit a dual role that induces apoptosis in tumor cells and paradoxically, DOX could activate a protection mechanism, preventing apoptosis [23-25].

On the other hand, senescence has recently been considered as another form of tumor cell response to chemotherapy [26,27]. This cellular state is considered a general biological program of growth permanent arrest and can be induced by telomere shortening (aging) or by injuries to DNA, such as those induced by chemotherapy, which do not involve telomere shortening (accelerated senescence). In this state, tumor cells cannot replicate [28-30]. Senescence was initially considered to be a protector mechanism against the development of neoplasms [28].

The aim of the present work was to study proliferation, viability, apoptosis, caspase-3, -8, and −9 activity, cytochrome *c* release, mitochondrial membrane potential (ΔΨm), senescence, p65 phosphorylation (NF-кB subunit), the Bcl-2 and Bcl-XL antiapoptotic proteins, and related genes induced by DOX and/or by the MG132 proteasome inhibitor in U937 leukemia cells.

## Results

### Early reduction of viability in U937 leukemia cells by MG132 + DOX treatment

**Figure 1:**
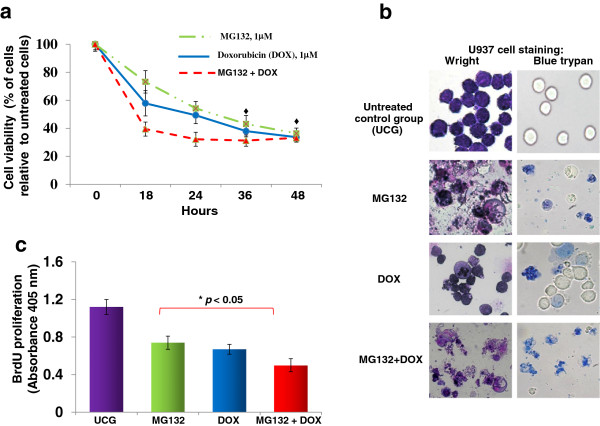
**MG132 + DOX induces a decrease in viability and proliferation in U937 cells.** U937 cells were treated with MG132 proteasome inhibitor (1 μM), Doxorubicin (DOX) 1 μM, and MG132 + DOX. U937 cells (2 × 10^4^) were incubated in the presence of different treatments for 18, 24, 36, and 48 h at 37°C in a humid atmosphere containing 5% CO_2_ and 95% air in RPMI-S culture medium. Subsequently, WST-1 was added and the cells were incubated for 3 h; then, viability was assessed by measuring the Optical density (OD) at 490 nm. The OD value of the Untreated control group (UCG) was taken as 100% cell viability **(a)**. After 24 h, morphological changes were observed, U937 cells were stained with blue trypan or fixed and stained with Wright on cover-glass slides and observed under a light microscope with zoom lens of 4X to 40X using a Leica DMLB microscope **(b)**. After 72 h, proliferation was assessed using BrdU **(c)**. The results represent the mean ± the Standard deviation (SD) of three independent experiments performed in triplicate. Statistical analysis, the Mann–Whitney *U* test. *p <0.05 MG132, DOX, or MG132 + DOX *vs* the Untreated control group (UCG); ●*p* <0.05 MG132 + DOX *vs* all groups; *♦p* <0.05 DOX, MG132, or MG132 + DOX *vs* the UCG.

First, the U937 cells were evaluated for viability by carrying out a dose–response effect and kinetics with the different treatments. As depicted in Figure 1a, an important toxicity effect was observed 18 and 24 h post-treatment, mainly in the MG132 + DOX-treated group. Viability was 39.4 ± 5.2% and 32.2 ± 4.5%, respectively (*p* <0.05) in comparison with that of all groups. After 36 and 48 h post-treatment, no differences were observed between the groups. Likewise we observed morphological changes in cells treated with MG132, DOX or MG132 + DOX (Figure 1b). We can observe that these treatments induce multi-lobular nuclei, increased cytoplasmic volume, and membrane blebbing, suggesting that U937 leukemic cells show signs of morphological membrane damage and apoptosis. Taken together, these results clearly confirm the toxic effect exerted by MG132 and the sensitization of U937 leukemia cells to the toxic action of DOX.

### MG132 + DOX induces a decrease in U937 leukemia cell proliferation

We evaluated the effect on the proliferation of U937 leukemia cells treated with MG132, DOX, or their combination (Figure 1c). We observed that in cells treated with MG132 or DOX, proliferation decreased in comparison with that of the Untreated control group (UCG) (*p* <0.05). However, it is important to stress that the cells treated with the combination of both drugs showed lower proliferation than those treated with each drug individually (*p* <0.05) (OD = MG132, 0.75 ± 0.06; DOX, 0.67 ± 0.05; and MG132 + DOX, 0.51 ± 0.06; *p* <0.05 vs. the UCG, 1.13 ± 0.09).

### Evaluation of apoptosis and caspase-3, -8, and −9 activation

**Figure 2:**
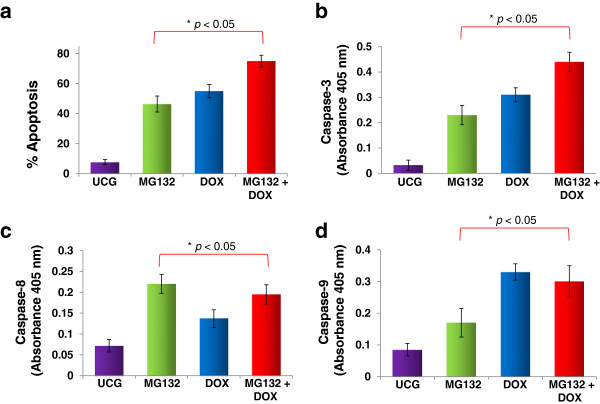
**Apoptosis and caspase activity in U937 cells treated with Doxorubicin (DOX), MG132, or MG132 + DOX.** U937 cells were incubated alone or in combination with MG132 (1 μM), DOX 1 μM, or MG132 + DOX for 24 h at 37°C in a humid atmosphere containing 5% CO_2_ in RPMI-S culture medium. After incubation, the cells were washed and apoptosis was assessed by flow cytometry using Annexin V-fluorescein isothiocyanate (FITC) **(a)**. For each sample, at least 20,000 events were acquired in a FACSAria-I cell sorter and the data were analyzed with FACSDiva software. The activity of caspase-3 **(b)**, -8 **(c)**, and −9 **(d)** was evaluated utilizing a caspase colorimetric staining kit. The results represent the mean ± the Standard deviation (SD) of three independent experiments performed in triplicate. Statistical analysis was performed by means of the Mann–Whitney *U* test. *●p* <0.05 MG132 + DOX *vs* MG132, DOX, or the Untreated control group (UCG); *p <0.05 all groups *vs* the UCG.

At 24 h post-treatment, apoptosis was evaluated in U937 cells treated with the different schedules. In Figure 2a, we can observe that the UCG showed a lower percentage of apoptosis (7.8 ± 1.6%) compared with that of the groups treated exclusively with MG132 or with DOX (45.4 ± 5.3% and 55.1 ± 5.4% of apoptosis, respectively; *p* <0.05). Interestingly, cell cultures exposed to MG132 + DOX exhibited superior values of apoptosis in comparison with the cells treated only with one drug, with a percentage of apoptosis of 75.1 ± 0.5% (*p* <0.05 when comparing MG132 + DOX vs. MG132- or DOX-treated cells), with an average increase for the three groups representing ∆% = 1017 in relation to the UCG. In order to confirm caspase participation, we evaluated caspase activity; in Figure 2b, we are able to observe that MG132 proteasome inhibitor and DOX-induced caspase-3 activity (*p* <0.05) in comparison with the UCG. Therefore, the MG132 + DOX group demonstrated most important induction of caspase-3 activity (*p* <0.05) in comparison with that of the other groups. In Figure 2c, we observe that the MG132, DOX, and MG132 + DOX groups induced caspase-8 activity in comparison with the UCG (*p* <0.05), the highest induction noted in the groups treated with MG132 or MG132 + DOX. In Figure 2d, we can also observe that the highest activity of caspase-9 was observed in DOX- or MG132 + DOX-treated groups (*p* <0.05).

### MG132 Proteasome inhibitor and DOX induce cleavage in caspase-3 and −9 and cytochrome *c* release

**Figure 3:**
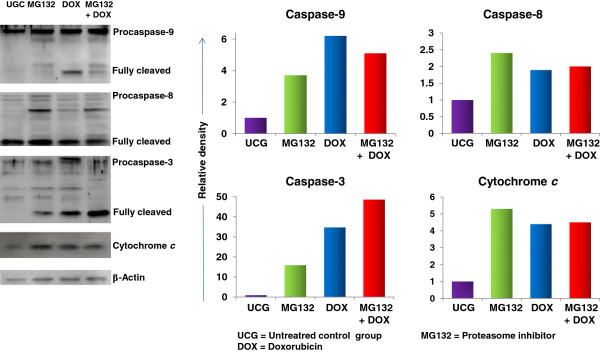
**Western blot analysis of caspases −3, -8, and −9 and cytochrome*c*in U937 cells treated with MG132, Doxorubicin (DOX) or MG132 + DOX.** U937 cells were cultured and treated with MG132 (1 μM), DOX 1 μM, or with MG132 + DOX. After 24 h, the cells were harvested and lysed. Equivalent amounts of individual lysates were placed onto 10% SDS gradient polyacrylamide gels for electrophoresis and then were electrotransferred onto Immobilon-P PVDF membranes. A representative study is shown. Relative density was calculated using IMAGEJ ver.1.46r software.

To confirm our results, we determined cleavage in caspases and in cytochrome *c* release. The results in Figure 3 allow us to observe that cell treatment with the MG132 proteasome inhibitor increased the cleavage of caspase-9 (3.7-fold), caspase-8 (2.4-fold), and of caspase-3 (15.9-fold), and also the release of cytochrome *c* (5.3-fold) compared with the UCG. Similarly, DOX treatment increased the cleavage of caspase-9 by 6.2-fold, of caspase-8 by 1.9-fold, and of caspase-3 by 34.7-fold, and cytochrome *c* release by 4.4-fold compared with that of the UCG. It is noteworthy that when we used MG132 + DOX, we observed considerably more cleavage of caspase-3 (48.5-fold) compared with MG132 or DOX alone and also when compared with the UCG. Additionally, when we used both drugs simultaneously, we observed an increase in cytochrome *c* release (4.5-fold) and the cleavage of caspase-8 (2-fold) and of caspase-9 (5.1-fold) in comparison with the UCG. These results show the sensitization of leukemia cells to chemotherapy induced by the MG132 proteasome inhibitor and demonstrates that caspases possess significant participation.

### MG132 + DOX induces potential mitochondrial membrane loss

**Figure 4:**
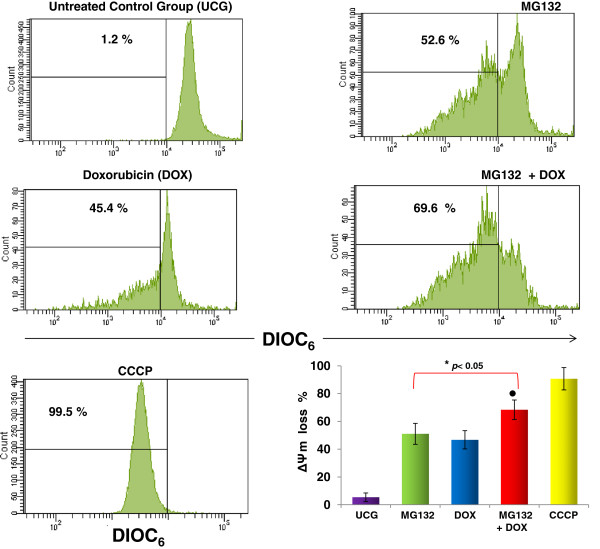
**MG132 + DOX induces potential mitochondrial membrane loss.** U937 cells were cultured and treated with MG132 (1 μM), DOX 1 μM, or MG132 + DOX. After 24 h, the cells were harvested and the ΔΨm was assessed by flow cytometry using DIOC_6_ staining. Protonophore Cyanide m-chorophenylhydrazone (CCCP) was used as a positive control. Representative histograms of each treatment are displayed. For each sample, at least 20,000 events were acquired in a FACSAria-I cell sorter and the data were analyzed with FACSDiva software. The graph shows the results as mean ± the Standard deviation (SD) of three independent experiments performed in triplicate. Statistical analysis, the Mann–Whitney *U* test. *p <0.05 all groups *vs* the Untreated control group (UGC); *●p* <0.05 MG132 + DOX *vs* all groups.

Because we observed cytochrome *c* release, it was in our interest to determine the ΔΨm in U937 leukemia cells treated with MG132, DOX, or MG132 + DOX; the results are represented in Figure 4. The ΔΨm did not change in the UCG. However, when the cells were treated with either MG132 or with DOX, an important loss of the ΔΨm was observed (52.1 ± 7.9% and 46.8 ± 6.6%, respectively; (*p* <0.05) compared with the UCG), and it is interesting that MG132 + DOX induces a more obvious ΔΨm loss in U937 cells (69.3 ± 6.9%) in comparison with that of the remaining groups (*p* <0.05).

### MG132 reduces DOX-induced senescence in U937 leukemia cells

**Figure 5:**
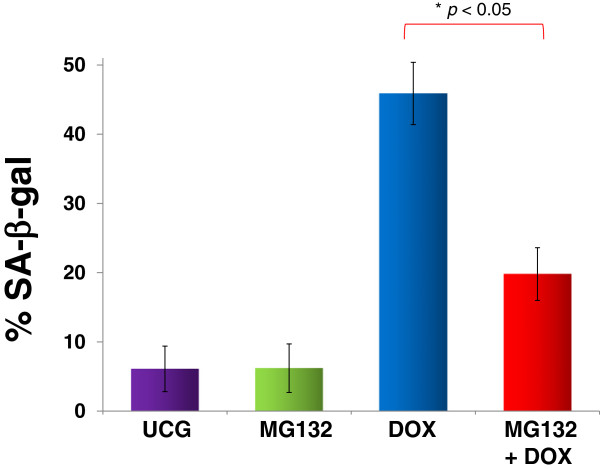
**Assessment of senescence in U397 human leukemic cells treated with MG132 (1 μM), Doxorubicin (DOX) 1 μM, or MG132 + DOX.** U937 cells (1 × 10^6^) were incubated alone or with different treatments for 24 h at 37°C in a humid atmosphere containing 5% CO2 and 95% air in RPMI-S medium. After incubation, the cells were washed in the same medium, and senescence was determined by measuring SA-β-gal utilizing flow cytometry. For each sample, at least 20,000 events were acquired in a FACSAria-I cell sorter and the data were analyzed with FACSDiva software. Results are shown as % and represent the mean ± the Standard deviation (SD) of three independent experiments performed in triplicate. Statistical analysis, the Mann–Whitney *U* test. *p <0.05 MG132 + DOX *vs* DOX; *♦p* <0.05 DOX *vs* all groups. UCG = Untreated control group.

It is well known that cells in senescence are SA-β-gal-positive; in Figure 5, we can observe that U937 leukemia-cell cultures, either without treatment or treated with MG132 alone, were practically non SA-β-gal-positive (6.2 ± 3.8% and 6.8 ± 3.2%, respectively); in contrast, DOX-treated cells exhibited a higher percentage of SA-β-gal-positive cells (45.8 ± 4.5%, respectively). However, when U937 cells were treated with MG132 + DOX, we observed a very important reduction in senescence (19.8 ± 3.8%; *p* <0.05) in comparison with cells treated only with DOX.

### Determination of p65 phosphorylation (NF-кB subunit) and Bcl-2 and Bcl-XL antiapoptotic proteins by flow cytometry

**Figure 6:**
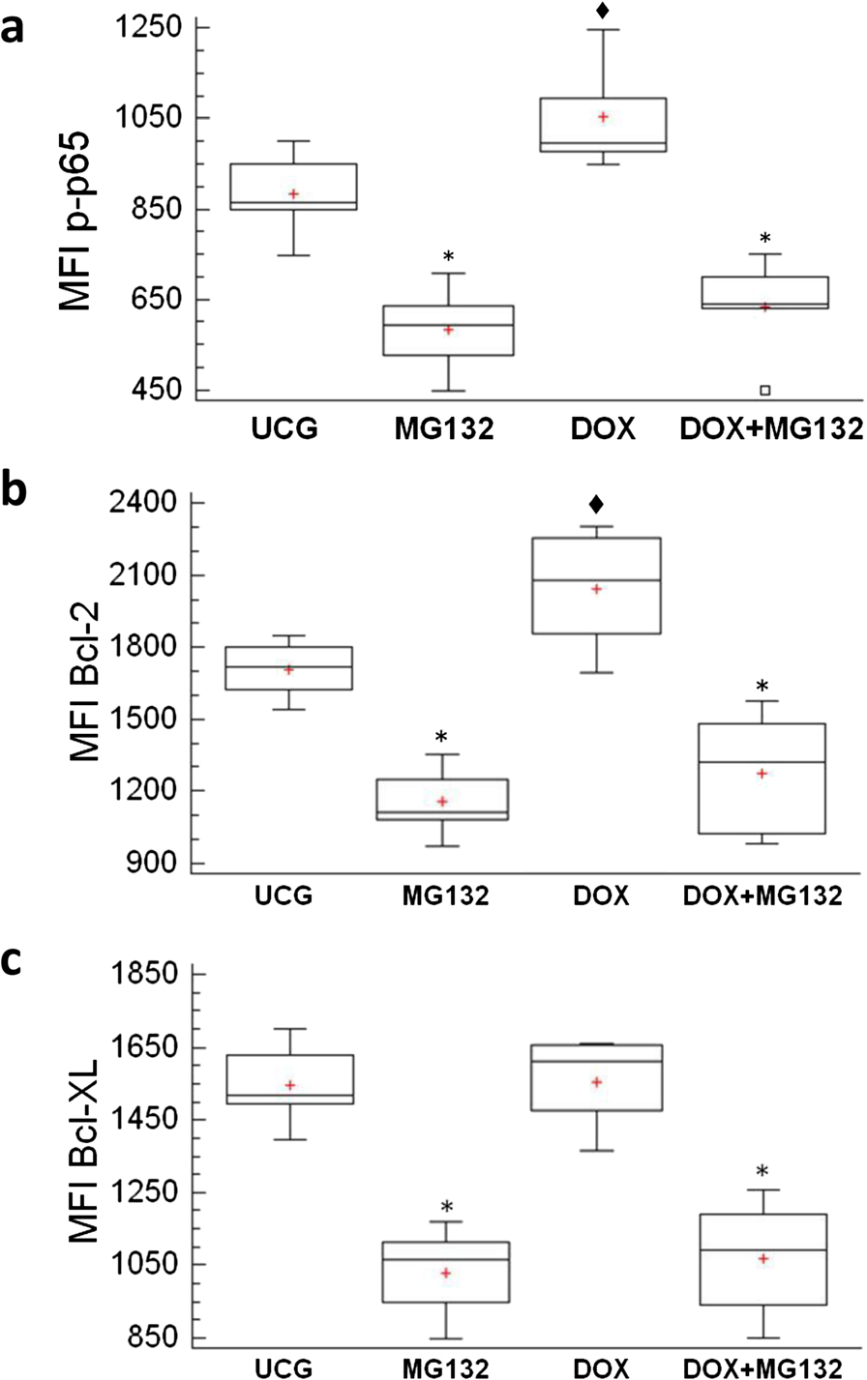
**Determination of phosphorylated p65 (NF-кB subunit), Bcl-2 and bcl-XL in U937 human leukemic cells treated*in vitro*with Doxorubicin (DOX), the MG132 proteasome inhibitor, and MG132 + DOX.** U937 cells were incubated, alone or in combination, with DOX 1 μM, MG132 (1 μM), or MG132 + DOX. After 1 h, the cells were harvested and the phosphorylated p65 protein was determined by flow cytometry **(a)**. U937 were treated with MG132, DOX or it´s combination after that the cells were harvested and the Bcl-2 **(b)** and Bcl-XL **(c)** antiapoptotic proteins were determined by flow cytometry. An appropriate isotype control was utilized to adjust background fluorescence. The graphs show the Mean fluorescence intensity (MFI) of p65, Bcl-2 or Bcl-XL. For each sample, at least 20,000 events were acquired in a FACSAria-I cell sorter and the data were analyzed with FACSDiva software. Results represent the mean ± the Standard deviation (SD) of three independent experiments carried out in triplicate. *♦p* <0.05 DOX *vs* the Untreated control group (UCG); *p <0.05 MG132 + DOX or MG132 *vs* DOX or the UCG.

As illustrated in Figure 6a, we are able to observe that DOX-treated U937 human leukemia cells increase p65 phosphorylation in comparison with the other groups (*p* <0.05). The MG132 proteasome inhibitor alone decreases this phosphorylation compared with the UCG (*p* <0.05). However, it is noteworthy that MG132 significantly reduces DOX-induced p65 phosphorylation in U937 leukemia cells (*p* <0.05) *vs* the DOX-treated group.

As depicted in Figure 6b, the Bcl-2 antiapoptotic protein is induced by DOX (*p* <0.05) *vs* the UCG; in contrast, DOX did not modify the Bcl-XL protein cell concentration (Figure 6c). In U937 leukemia cells treated with MG132 or MG132 + DOX, the behavior is comparable; Bcl-2 and Bcl-XL antiapoptotic proteins are significantly reduced in comparison with those of the UCG and the DOX-treated group (*p* <0.05).

### Changes in the expression of proapoptotic, antiapoptotic, and NF-кB-related genes

**Figure 7:**
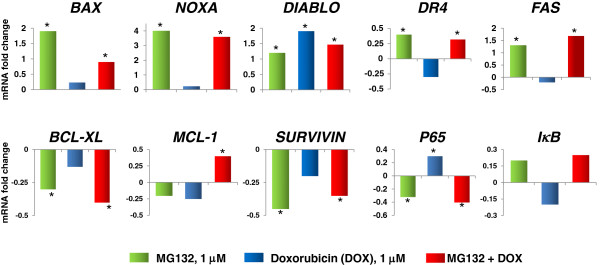
**Expression of pro- and antiapoptotic genes in U937 human leukemic cells treated with Doxorubicin (DOX), MG132, and MG132 + DOX.** U937 human leukemia cells were incubated, alone or in combination, with DOX 1 μM, MG132 (1 μM), or MG132 + DOX for 3 h. Gene expression was assessed by quantitative real-time PCR. Data are expressed as relative mRNA fold-change. *RPL32* were used for normalization, and the value of untreated cells, as calibrator. Variations were considered significant at ≥30% compared with the constitutive gene. In all cases, the Standard deviation (SD) was not >0.08.

We employed real-time PCR to determine mRNA expression (Figure 7). In DOX-treated U937 cell cultures, we found upregulation of the *DIABLO* and *p65* genes in comparison with untreated cells (*p* <0.05); the most important upregulation was observed with *DIABLO* (a 1.7-fold upregulation). Likewise, MG132 induced downregulation of the *BCL-XL* and *SURVIVIN* antiapoptotic genes and of the *p65* NF-кB-related gene (*p* <0.05) *vs* untreated cells. We also observed upregulation of the *BAX*, *NOXA*, *DIABLO*, *DR4*, and *FAS* genes when the cells were treated with MG132 (*p* <0.05). In the case of U937 cells treated with MG132 + DOX, the proapoptotic genes *BAX*, *NOXA*, *DIABLO*, *DR4,* and *FAS* were upregulated (*p* <0.05); the highest upregulation was observed in *DIABLO and FAS* expression (1.68- and 1.47-fold upregulation, respectively). In MG132 + DOX-treated U937 cells, we observed strong downregulation in the *BCL-XL* and *SURVIVIN* antiapoptotic genes and in *p65* NF-кB-related gene (*p* <0.05). In general, the data obtained suggested that MG132 + DOX treatment of U937 human leukemia cells favors the activation of genes with proapoptotic activity.

## Discussion

In this work, we demonstrated that the combination MG132 + DOX can induce an important reduction in U937 cells proliferation. This is crucial because leukemia cells could lose the ability to continue proliferating, thus the opportunity to cause more damage. This can be explained by the viability reduction and the apoptosis observed when U937 cells were treated with both drugs. Employing the WST-1 spectrophotometric assay, we determined viability in U937 cells at different times. The three compounds exhibited different kinetics, and after 18 and 24 h of cell exposure, the highest time was with the MG132 + DOX treatment. However, these differences only indicate a difference in kinetics, because at 36 and 48 h, cell viability is comparable among the three research groups at 60% toxicity; however, the difference in the kinetics suggests the specificity of the treatments. It is interesting that determination of apoptosis exhibited the same behavior between MG132 and DOX; both compounds induce important percentages of apoptosis; however, this was more important in the MG132 + DOX group (*p* <0.05). We observed by light microscopy that MG132, DOX, and MG132 + DOX induce membrane and nuclei damage in U937 leukemic cells. The morphology is consistent with the change observed in viability with WST-1 and apoptosis with annexin-V by flow cytometry. Taken together, these results show that the MG132 proteasome inhibitor can sensitize U937 leukemia cells to DOX-induced apoptosis.

It is noteworthy that antitumoral drugs cannot differentiate between reproducing cells of normal tissues and cancer cells, and unfortunately normal cells are damaged; this results in side effects, hence the importance of understanding how antitumor drugs work, and this will allow finding a balance between destroying the cancer cells without impacting normal cells.

It has been reported that the MG132 proteasome inhibitor affect normal cells less than leukemic cells, and that normal cells can survive after being submitted to therapeutic doses of proteasome inhibitors [31,32]. Therapeutic doses of proteasome inhibitors only inhibit chymotrypsin sites in the 20S proteasome, by which protein degradation decreases by about one half, that is has a limited extension, and that trypsin-like and caspase-like remain functioning [33,34]. In this sense, healthy cells have a range of proliferation that is much lower than that of cancer cells; therefore, there is less need for proteasomal regulatory functions. Also, tumor cells exhibit an increase in proteasome proteolytic activity, and this can explain their increased susceptibility to apoptosis in cancer cells than in normal cells [35].

A similar effect is observed with DOX, because rapidly replicating cells, such as cancer cells, exhibit greater sensitivity to DOX, which results in more DNA damage than in normal cells; however DOX can also be toxic to healthy cells. DOX is used to treat non-Hodgkin lymphoma, multiple myeloma, acute leukemia, Kaposi’s sarcoma, breast cancers, adrenal cortex, endometrial, lung, and ovarian cancer [36]. However, its side effects are also well known. It has been reported that DOX induces apoptosis in endothelial and myocardial cells this mainly due to H_2_O_2_ production [37]. In leukemic and other tumor cells, it has been observed that DOX can intercalate with DNA, inducing histone eviction from open chromosomal areas and taking part in oxidation-reduction reactions [38-40] and a mechanism has been disclosed by which DOX induces apoptosis in tumor cells, consisting of the activation of the factor CREB3L1, which activates transcription of genes that disrupt the cell cycle [41]. Induction of apoptosis in U937 leukemia cells by means of DOX is related with caspase activation, as has been shown in other tumor cells [42,43]. In the present study, we found an important induction of caspase-3, -8, and −9 activity when U937 cells were treated with MG132, DOX, or MG132 + DOX. Although it has been published that the MG132 proteasome inhibitor induces apoptosis via deregulation of the endoplasmic reticulum in the cell [14] or by induced cell cycle arrest [44], in our study we observed a higher increase in caspase-8 when U937 cells were treated with MG132, whereas in that with DOX, we observed an increase in caspase-9. In this regard, it has been observed that the MG132 proteasome inhibitor prevents degradation of the cleaved forms of caspase-8, and this process can be specified for the activated form of caspase-8 [45]. The effect observed in caspase 9 when U937 cells were treated with DOX can be explained by the induction of free radicals by DOX, which destabilizes mitochondria, facilitating the release of cytochrome *c* and the activation of casapase-9, as has been observed in other studies [46,47]. However, it is important to stress that when we used MG132 + DOX, we observed increased activity in relation to caspase-3 and its cleavage, as observed in the Western blot. This observation coincides with the potential mitochondrial membrane loss observed when both drugs were combined and with the cytotoxicity assays.

Under physiological conditions, the mitochondria have a high ΔΨm, which is generated by the respiratory chain and which facilitates the generation of ATP. In response to some proapoptotic signals, including ROS and calcium accumulation, there is an increase in conductance in the so-called Permeability transition pore complex (PTPC), which allows the entry of small molecules into the mitochondrial matrix. This transition in mitochondrial permeability results in immediate ΔΨm loss, which culminates in the release into the cytosol of cytotoxic proteins that are normally confined within the mitochondrial intermembrane space [48]. Alterations in the structure and function of mitochondria and an increase in mitochondrial membrane potential have been reported in leukemic and tumors cells in comparison with normal cells [49,50]. Cancer cells have hyperpolarized their mitochondrial membranes compared with normal cells, which prevents them from throwing the switch of apoptosis regardless of age or mutation in their genes [50-52].

This may explain what we observed in our study, where MG132 and DOX or its combination induced ΔΨm loss and the release of cytochrome *c* was observed when U937 leukemia cells were treated. Importantly, when we treated leukemia cells with both drugs, we observed the greatest increase in the ΔΨm loss; this combination of drugs disrupts ΔΨm and facilitates the induction of apoptosis in these cells. Senescence is the mechanism whereby the cells can control cell division and stop uncontrolled growth [53,54]. However, in senescence, despite that the cells are not able to divide, they remain metabolically active and produce growth factors that can promote pre-tumor or tumor-cell growth and proliferation [55-57]. It is very interesting that in our study, as expected, DOX induced senescence in U937 leukemia cells; conversely, the proteasome inhibitor MG132 possessed did not exert an effect on this process. In addition, when both drugs were combined, we observed a decrease of senescence compared with cells treated with DOX alone; however, senescence remained significantly higher compared with that of the untreated group. It is noteworthy that other products not classified as antitumor drugs, such as Pentoxifylline (PTX), which are used experimentally to sensitize tumor cells to chemotherapy, exhibit the same behavior [58]. Whether cellular senescence induced by antitumor therapy can act as friend or foe is difficult to determine precisely because recent data indicate that factors secreted by senescent cells can also alter the microenvironment and enhance the tumor growth of neighboring tumor cells, indicating that this protective mechanism can act as a double-edged sword [59,60]. At any rate, the treatment schedule with the two compounds shows a clear advantage because after treatment, the genetic balance is inclined to trigger the apoptotic process rather than senescence.

On the other hand, it is well known that NF-кB is responsible for the activation of genes involved in proliferation and tumor survival, such as the apoptotic proteins Bcl-2 and Bcl-XL [61,62]. In fact, this transcription factor has been found to be overactivated in many tumors [63-65]. It has been published that proteasome inhibition sensitizes glioblastoma cells to Tumor necrosis factor (TNF)-related apoptosis-inducing ligand (TRAIL)-induced apoptosis by the NF-кB independent pathway [66]. In this respect, other studies show that MG132 proteasome inhibition induces apoptosis in glioblastoma cells through inhibition of the PI3K/Akt and NF-кB pathways [67], and that this inhibitor disrupts DOX-induced NF-кB activation in DOX-resistant K562 erythromyeloblastoid leukemia cells [68]. These differences and similarities observed show that although tumor cells share many antitumor resistance pathways, each cell can respond differently to an antitumor agent or to its possible combinations with other drugs. In our study, we observed that DOX induces the phosphorylation of *p65* (NF-кB subunit), which is consistent with other studies, which have found that DOX can induce NF-кB activation, as in breast cancer [69] and as in acute myelogenous leukemia [70], and cervical cancer [71]. This may help explain resistance to cancer therapies, because these drugs can turn on the NF-кB pathway, as observed for other drugs [72,73]. In this regard, it has been observed that DOX in therapeutic doses can increase proteasome activity, thus favoring the activation of transcription factors such as NF-кB. This observation may be due to that DOX could change the conformation of the proteasome catalytic sites, rendering these more efficient [11]; however, it is noteworthy that when the MG132 proteasome inhibitor is used with DOX, this combination induces *p65* gene downregulation and decreases p65 protein phosphorylation, reaching similar values when using MG132 alone.

Tumor cells develop resistance to apoptosis through multiples mechanisms, including the expression of antiapoptotic proteins [74]. In this regard, proteins Bcl-2 and Bcl-XL have been found overexpressed in several tumors [75]. We observed that DOX induces Bcl-2 overexpression in U937 leukemic cells and in contrast, the proteasome inhibitor MG132 decreases Bcl-2 baseline levels and in turn reduces DOX-induced Bcl-2 overexpression. Similar behavior was observed in terms of Bcl-XL in cells treated with the proteasome inhibitor MG132. It is noteworthy that NF-кB is involved in the regulation of Bcl-2 and Bcl-XL proteins, and the findings in our study highlight the importance of the fact that the MG132 proteasome inhibitor alone or in combination with DOX not only reduces p65 (NF-kB subunit) phosphorylation, but also simultaneously downregulated at the Bcl-2 and Bcl-XL antiapoptotic proteins, which aid in sensitizing DOX-treated U937 human leukemic cells to apoptosis.

Another target of the treatments are the *I*к*B* and *p65* genes. We observed that DOX induces upregulation of the *p65* gene, and that MG132, alone or in combination with DOX, downregulated *p65*. This is important because the *p65* gene regulated the p65 protein (NF-кB subunit), which increases NF-кB availability in the cytoplasm, and this can promote survival in cancer cells [76].

It is important to note that several genes that are modulated by NF-кB were downregulated by treatments particularly *BCL-XL* and *SURVIVIN* genes when cells were treated with the MG132 proteasome inhibitor and MG132 + DOX. An NF-кB overactivation has been observed in cancer, and this leads to upregulation of the *BCL-XL, SURVIVN* and *MCL-1* genes, and this increase resistance to apoptosis in tumor cells [77]. Bcl-XL suppresses the release of cytochrome *c* from the mitochondria, survivin interferes with caspase 9 activation in tumor cells and Bcl-XL, Mcl-1 and survivin can antagonize the proapoptotic effect of bax and Diablo in tumor cells [78,79]. It is interesting to stress that downmodulation of *BCL-XL*, *MCL-1* and *SURVIVIN* genes by the treatments coincides with an increase in the release of cytochrome *c*, and caspase-9 activity and with upregulation of the BAX and DIABLO genes observed when U937 leukemic cells were treated with MG132, DOX or their combination.

In this manner, by inhibiting the aforementioned genes, there is less cytoplasmic availability of NF-кB, thus there is less opportunity to activate survival programs [80]. With the combination of both drugs, we observed a significant trend toward upregulation of proapoptotic genes *DIABLO*, *NOXA*, *BAX*, *FAS*, and *DR4*, and downregulation of the antiapoptotic genes *BCL-XL* and *SURVIVIN*. This demonstrates that the MG132 proteasome inhibitor in combination with DOX can induce a balance in favor of the proapoptotic machinery in U937 human leukemic cells, and this is in agreement with our previous observations and confirms the concept of chemotherapy with rational molecular bases.

## Conclusion

Our results show that the MG132 proteasome inhibitor can sensitize U937 leukemic cells to the toxic action of DOX with a greater apoptotic tendency.

## Methods

### Cells

We employed the cell line U937 (ATCC CRL-1593.2) that is derived from human monocytic leukemia. These cells were maintained under cryopreservation and cultivated in Roswell Park Memorial Institute (RPMI)-1640 medium (GIBCO, Invitrogen Corp., Carlsbad, CA, USA), to which we added 10% Fetal bovine serum (FBS) (GIBCO), L-glutamine to a final concentration of 2 mM (GIBCO), and antibiotics (GIBCO), which will be designated RPMI-S. Cells were maintained at 37°C in a humid atmosphere containing 5% CO_2_ and 95% air.

### Drugs

DOX was obtained from Pisa Laboratories, México and stored at −4°C for use in the experiment in <4 days. The desired concentration was adjusted in the RPMI-1640 culture medium immediately prior to use.

With regard to the proteasome inhibitor, we utilized MG132 (N-CBZ-LEU-LEU-AL) (Sigma-Aldrich, St. Louis, MO, USA). Five mg were dissolved in 250 μL of Dimethyl sulfoxide (DMSO; Sigma-Aldrich), dividing it into 25-μL aliquots and stored at −20°C. Immediately prior to its use, the inhibitor was diluted in the RPMI-1640 culture medium, adding 5 μL to the cultures to obtain a final concentration of 1 μM.

### Cell culture and experimental conditions

U937 cells (2.5 × 10^5^/mL in T75 flasks) were grown in RPMI-S for 24 h and collected by centrifugation. Cells were reseeded in 24-well plates, and 1 × 10^6^ cells were treated either with DOX (1 μM), or MG132 (1 μM), or MG132 + DOX (final concentrations). After 24 h, morphological changes were observed and cells were harvested and apoptosis, caspase cleavage or activity, ΔΨm loss, senescence, and Bcl2- and Bcl-XL antiapoptotic proteins were determined. *p65* phosphorylation was analyzed 1 h after treatment with DOX or with MG132. For the gene-expression study, cells were incubated for 3 h with the drugs. For viability determination, U937 cells were reseeded in 96-well plates, and 2 × 10^4^ cells were treated with DOX, MG132, or both; viability was measured at 18, 24, 36, and 48 h of culture and proliferation at 72 h. Concentrations of the treatments employed in this study were previously confirmed as being the most favorable for the induction of apoptosis in this experimental model [7].

### Morphological changes in U937 treated with MG132 or Doxorubicin

U937 cells were treated with the MG132 proteasome inhibitor, Doxorubicin or both drugs. Subsequently, morphological changes in U937 cells treated or not were either microscopically observed in cells stained with blue trypan or fixed and stained with Wright on cover-glass slides and observed under a light microscope with zoom lens of 4X to 40X using a Leica DMLB microscope (Leica Microsystems, Wetzlar, Germany). The photographs were taken with a digital camera (Olympus C5060).

### Proliferation by BrdU

Proliferation was determined with the 5-Bromo-2’-deoxy-uridine (BrdU) Labeling and Detection Kit III assay (Roche, Mannheim, Germany) following the manufacturer’s instructions. Briefly, the U937 cells were cultured. After 72 h, we added BrdU labeling solution overnight; then, the cells were air dried for 2 h at 60°C. Later, we added precooled fixative solution for 30 min and the nucleases were added for 30 min at 37°C. Subsequently, anti-BrdU-POD was added for 30 min, and finally peroxidase ABTS peroxidase substrates were added for 30 min. Afterward, proliferation was determined in a microplate reader (Synergy™ HT Multi-Mode Microplate Reader; Biotek, Winooski, VT, USA) by spectrophotometry at 405 nm with a 490-nm reference wavelength. Data are reported as the mean ± standard deviation (SD) of the optical density (OD) values obtained for each group.

### Cell viability

Cell viability was determined with the WST-1 assay (BioVision Research Products, Mountain View, CA, USA) following the manufacturer’s instructions; this study is based on the reduction of Tetrazolium salts (WST-1) to formazan; after 18, 24, 36, and 48 h, WST-1 was added and the cells were incubated for 3 h. Then, cell viability was determined in a microplate reader (Synergy™ HT Multi-Mode Microplate Reader; Biotek) by spectrophotometry at 490 nm. Data are reported as the percentage of cell viability in comparison to that of its respective non-treated control group (100%).

### Assessment of apoptosis, ΔΨm loss, and senescence by flow cytometry

Apoptosis was evaluated by means of the annexin V-Fluorescein isothiocyanate (FITC) test (Annexin-V-Fluos; Roche). U937 cells (1 × 10^6^) were incubated for 15 min with annexin V-Fluorescein isothiocyanate (FITC) according to annexin-V-Fluos kit instructions. Annexin V-FITC cells were considered to be undergoing apoptosis and those negative for FITC were considered to be alive. For assessment of ΔΨm loss, after culture, 1 × 10^6^ U937 cells were washed twice with PBS, resuspended in 500 μL of PBS containing 20 nM of 3,3-dihexyloxacarbocyanine iodide (DIOC6; Sigma-Aldrich), and then incubated at 37°C for 15 min. The percentage of cells with ΔΨm loss was analyzed by flow cytometry. As an internal control of the disrupted ΔΨm, cells were treated for 4 h with 150 μM of protonophore Carbonyl cyanide m-chlorophenyldydrazone (CCCP, Sigma-Aldrich) positive control. Determination of senescence was performed by measuring β-galactosidase activity (senescence-associated beta-gal, SA-β-gal). After 24 h of incubation, 100 nM of Bafilomycin A1 (Sigma-Aldrich) was added and the cells were incubated for 1 h; then, 10 μM of C12FDG (fluorogenic glycosidase substrate; Invitrogen Corp., Carlsbad, CA, USA) was added, and the mixture was incubated for 15 min. Finally, the cells were harvested, washed twice with PBS, and resuspended in PBS before being analyzed by flow cytometry. Results are represented as the percentage of apoptosis, ΔΨm loss, and senescence. At least 20,000 events were acquired for each sample in a FACSAria-I cell sorter (BD Biosciences, San Jose, CA, USA) and the data were processed with FACSDiva software (BD Biosciences).

### Assessment of caspase-3, -8, and −9 activity

Caspase activity was determined with the caspase −3, -8 and −9 colorimetric kits (BioVision Research Products). U937 cells (1 × 10^6^) were treated for 24 h with the set of drugs; afterward, the cells were washed twice with PBS and then were resuspended in cell lysis buffer and incubated on ice for 10 min. Crude lysates were centrifuged and supernatant was transferred to a fresh tube. Then we added 2X reaction buffer containing 10 mM DTT and 5 mL of the 4-mM substrate of caspase-3, -8, or −9 and incubated this at 37°C for 1 h. Subsequently, absorbance was measured in a microplate reader (Synergy HT Multi-Mode Microplate Reader; Biotek) at 405 nm. Data are reported as the mean ± SD of the OD values obtained in each group.

### Protein extraction for caspase-3, -8, and −9 and cytochrome *c* and Western blot assay

U937 cells (5 × 10^6^) cells were treated with MG132, DOX, or both drugs for 24 h. Afterward, the cells were harvested, washed twice with PBS, and lysed with RIPA buffer containing protein inhibitors. Following sonication (15-pulse, 505 ampl.), the protein extracts were obtained after 30 min of incubation at 4°C and 5 min of centrifugation at 14,000 rpm/4°C. Protein concentrations were determined utilizing the Dc Protein Kit (Bio-Rad Laboratories, Inc., Hercules, CA, USA). Total cell protein (40 μg) was subjected to electrophoresis employing a 10% Sodium dodecyl sulfate (SDS) polyacrylamide gel. Proteins were transferred onto Immobilon-P PVDF membranes (Millipore, Bedford, MA, USA) and incubated with 1X Western blocking reagent (Roche) during 1.5 h for nonspecific binding. Immunodetection of caspase-3, -8, and −9 was performed using anti-caspase-3, -8, and −9 antibodies (BioVision) and cytochrome *c* was effected using and anti-cytochrome *c* antibody (BioLegend, San Diego, CA, USA) at 4°C overnight. After incubation with a horseradish peroxidase-conjugated secondary antibody (Santa Cruz Biotechnology, Santa Cruz, CA, USA), immunoreactive proteins were visualized by Western blotting luminol reagent utilizing ChemiDocTM XRS equipment (Bio-Rad) with Quantity One 1-d Analysis software (Bio-Rad). β-Actin antibody (Santa Cruz Biotechnology) was used as a control. Protein levels in Western blot were quantified using the ImageJ 1.46r software package (NIH, Bethesda, MD, USA).

### Determination of the Bcl-2 and Bcl-XL antiapoptotic proteins and p65 phosphorylation by flow cytometry

For determination of Bcl-2, Bcl-XL and phosphorylated p65 in normal untreated and treated cell cultures, we used Alexa Fluor® 647 mouse anti-human Bcl-2 and Alexa Fluor® 647 mouse anti-human Bcl-XL proteins (Santa Cruz Technologies, Inc.) and Alexa Fluor® 647 mouse anti-human NF-кB p65 (pS529) (BD Biosciences) by flow cytometry and stained according to protocol to detect protein or activation of the phosphorylation state. An appropriate isotype control was utilized in each test to adjust background fluorescence, and results are represented as the Mean fluorescence intensity (MFI) of Bcl-2, Bcl-XL, and phosphorylated p65. At least 20,000 events were acquired for each sample in a FACSAria-I cell sorter (BD Biosciences) and the data were processed with FACSDiva software (BD Biosciences).

### Quantitative real-time PCR

Total RNA of the U937 cells was obtained after 3 h of incubation with the different treatments using the Purelink™ Micro-to-midi Purification System for total RNA (Invitrogen). The cDNA was synthesized starting from 5 μg of total RNA using the Superscript III First-Strand Synthesis Supermix kit (Invitrogen). Real-time PCR was carried out with the System Light Cycler® 2.0 (Roche Applied Science, Mannheim, Germany), for which we used a DNA Master plus SYBR Green I (Roche Applied Science). Analysis of the amplification curves of the PCR reactions was carried out with Light Cycler® software (Roche Applied Science). Data are presented in relative normalized quantities using the *RPL32* ribosomal gene expression as reference. The oligonucleotides (Invitrogen) were designed employing Oligo ver.6 software (Table 1) using the gene sequences reported in the National Information Biotechnology Center Nucleotide Data Base (http://www.ncbi.nlm.nih.gov).

**Table 1:** Primer pair used for real-time quantitative PCR

**Gene**	**Primer pair sequences**	**Gen Bank Accession No.**
*NOXA*	5'GAC ATG CCT GGG AAG AAG G3'	NM021127
	5'TCC TGA GCA GAA GAG TTT GGA 3'	
*BAX*	5'TTT GCT TCA GGG TTT CAT CC 3'	NM138764
	5'CAG TTG AAG TTG CCG TCA GA 3'	
*DIABLO*	5'TGA CTT CAA AAC ACC AAG AGT A3'	NM019887
	5'TTT CTG ACG GAG CTC TTC TA 3'	
*DR4*	5’CTC GCT GTC CAC TTT CGT CTCT3’	NM003844
	5’GTC AAA GGG CAC GAT GTT3’	
*FAS*	5’TGA ACA TGG AAT CAT CAA GGA3’	NM000043
	5’CAA AGC CTT TAA CTT GAC TT3’	
*BCL-XL*	5'GCA GGC GAC GAG TTT GAA CT 3'	NM138578
	5'GTG TCT GGT CAT TTC CGA CTG A 3'	
*MCL-1*	5'CAC GAG ACG GTC TTC CAA GGA TGC T 3'	NM021960
	5'CTA GGT TGC TAG GGT GCA ACT CTA GGA 3'	
*SURVIVIN*	5'TGA GCT GCA GGT TCC TTA TCT G 3'	NM001168
	5'GAA TGG CTT TGT GCT TAG TTT T 3'	
*IkBa*	5'GGA TAC CTG GAG GAT CAG ATT A 3'	NM001278
	5'CCA CCT TAG GGA GTA GTA GAT CAA T 3'	
*P65*	5'GCA GGC TCC TGT GCG TGT CT 3'	NM02975
	5'GGT GCT CAG GGA TGA CGT AAA G 3'	
*L32*	5'GCA TTG ACA ACA GGG TTC GTA G 3'	NM000994
*RIBOSOMAL*	5'ATT TAA ACA GAA AAC GTG CAC A 3'	
*PROTEIN*		

The oligonucleotides were designed using Oligo ver.6 software. Gene sequences were obtained from the GenBank Nucleotide Database of the National Center for Biotechnology Information (NCBI) (http://www.ncbi.nlm.nih.gov).

### Statistical analysis

All of the experiments were performed in triplicate and were repeated three times. The values represent the mean ± SD of the values obtained. Statistical analysis was carried out with the non-parametric Mann–Whitney *U* test, considering *p* <0.05 as significant. In some experiments, we calculated the ∆%, which represents the percentage of increase or diminution in relation to the non-treated control group. For the different genes, significant variation was considered at ≥30% compared with that of the constitutive genes.

## Competing interests

The author declares no potential conflict of interests.

## Authors’ contributions

PCO-L, AB-C, and GH-F designed and performed the research, analyzed the data, and drafted the manuscript; JML-D, JRD-R, PG-L, OG-R and RC-C performed some of the research and analyzed the data, and AA-L and LFJ-S conducted the molecular study and analyzed the data. All of the authors read and approved the final manuscript.
